# Design and simulation of CsPb._625_Zn._375_IBr_2_-based perovskite solar cells with different charge transport layers for efficiency enhancement

**DOI:** 10.1038/s41598-024-81797-x

**Published:** 2024-12-03

**Authors:** M. Khalid Hossain, Md Aminul Islam, M. Shihab Uddin, Prabhu Paramasivam, Junainah Abd Hamid, Razan A. Alshgari, V. K. Mishra, Rajesh Haldhar

**Affiliations:** 1 Institute of Electronics, Atomic Energy Research Establishment, Bangladesh Atomic Energy Commission, Dhaka, 1349 Bangladesh; 2 Department of Advanced Energy Engineering Science, Interdisciplinary Graduate School of Engineering Sciences, Kyushu University, Fukuoka, 816-8580 Japan; 3 School of Electrical, Computer and Energy Engineering, Arizona State University, Tempe, AZ 85281 USA; 4 Department of Electrical and Electronic Engineering, Islamic University, Kushtia, 7000 Bangladesh; 5 Centre for Research Impact & Outcome, Chitkara University Institute of Engineering and Technology, Chitkara University, Rajpura, Punjab 140401 India; 6 Department of Mechanical Engineering, Mattu University, Mettu, 318 Ethiopia; 7 Management and Science University, Shah Alam, Selangor Malaysia; 8 Chemistry Department, College of Science, King Saud University, Riyadh, 11451 Saudi Arabia; 9 School of Chemical Engineering, Yeungnam University, Gyeongsan, 38541 Republic of Korea

**Keywords:** Double perovskite solar cell, CsPb._625_Zn._375_IBr_2_ light absorber, ZnSe ETL, MoS_2_ HTL, SCAPS-1D, Materials science, Physics

## Abstract

In this work, CsPb._625_Zn._375_IBr_2_-based perovskite solar cells (PSCs) are numerically simulated and optimized under ideal lighting conditions using the SCAPS-1D simulator. We investigate how various hole transport layers (HTL) including Zn_3_P_2_, PTAA, MoS_2,_ MoO_3,_ MEH-PPV, GaAs, CuAlO_2_, Cu_2_Te, ZnTe, MoTe_2_, CMTS, CNTS, CZTS, CZTSe and electron transport layers (ETL) such as CdS, SnS_2_, ZnSe, PC_60_BM interact with the devices’ functionality. Following HTL material optimization, a maximum power conversion efficiency (PCE) of 16.59% was observed for the FTO/SnS_2_/CsPb._625_Zn._375_IBr_2_/MoS_2_/Au structure, with MoS_2_ proving to be a more economical option. The remainder of the investigation is done following the HTL optimization. We study how the performance of the PSC is affected by varying the materials of the ETL and to improve the PCE of the device, we finally optimized the thickness, charge carrier densities, and defect densities of the absorber, ETL, and HTL. In the end, the optimized arrangement produced a V_OC_ of 0.583 V, a J_SC_ of 43.95 mA/cm^2^, an FF of 82.17%, and a PCE of 21.05% for the FTO/ZnSe/CsPb._625_Zn._375_IBr_2_/MoS_2_/Au structure. We also examine the effects of temperature, shunt resistance, series resistance, generation rate, recombination rate, current-voltage (JV) curve, and quantum efficiency (QE) properties to learn more about the performance of the optimized device. At 300 K, the optimized device provides the highest thermal stability. Our research shows the promise of CsPb._625_Zn._375_IBr_2_-based PSCs and offers insightful information for further development and improvement.

## Introduction

Perovskite solar cells (PSCs) are regarded as one of the more viable variants for harnessing solar energy due to their appealing bandgap, high carrier mobility, and high absorption coefficient, among other desired optoelectronic attributes^1–4^. PSCs present a cost-effective and straightforward deposition method for harvesting solar energy compared to silicon solar cells^5–8^. Moreover, the power conversion efficiency (PCE) of PSCs has risen dramatically throughout the preceding 12 years, rising from 3.8 to 26.1%, enabling them to contend with silicon solar cells^9–11^.

The perovskite material has the chemical formula ABX_3_, where A represents an organic cation [CH_3_NH^3+^], B represents a divalent metal ion (Pb^2−^, Sn^2−^), and X (Br^−^, I^−^, Cl^−^) represents a halide ion when positioned at the body middle of the octahedron, six halide anions surround cation B, which has favorable bandgaps, high absorption coefficient, lingering carrier diffusion length, exalted carrier mobility, and superb carrier lifetime^12,13^. However, despite exhibiting excellent optoelectronic properties and evolutionary success in PCE within a very short time, the lack of lingering-term stability is still taken into account as the biggest hindrance to making PSCs commercially available^14^. Under the influence of UV light, redox reactions trigger chemical decomposition. Moreover, the polarity of water molecules adversely affects the hydrogen bonds of perovskites^15,16^. As a result, the stability of PSCs decreases when they are exposed to moisture, UV light, and the surrounding environment. This leads to an inevitable deterioration in PSC performance within a few weeks^15–17^. Therefore, improving the stability of perovskite has taken special attention in recent years so that stable PSCs can be achieved without compromising performance.

Different strategies are investigated to address these issues and improve perovskite stability. Two-dimensional (2D) perovskite and inorganic perovskite are possible solutions to the stability issues^18–20^. However, 2D perovskite is not an ideal option for developing high-performance PSCs due to its huge bandgaps, asymmetric crystallographic orientations, as well as lengthy organic cation layers, which limit high carrier generation and slow effective charge carrier separation^12,21^. So, inorganic perovskite materials can be used in solar cells to improve the PSC’s steadiness and retain their efficiency^22,23^. Cesium ions (Cs^+^) can be used as a replacement for organic cations to enhance the resilience of materials composed of perovskites^20^. In 2012, Cs-based CsSnI_3_ perovskite was introduced, achieving an efficiency of 0.88%^24^. Since then, many cesium lead halide perovskites have been formed to be utilized in perovskite solar cells (PSCs), including CsPbI_3_, CsPbBr_3_, and CsPbCl_3_^25–29^. CsPbI_3_ has demonstrated a maximum PCE of ∼19% as of 2021^30^. The PCE of CsPbX_3_-based devices is still lower than that of their organic-inorganic counterparts, even with the notable advancements in this area. So, to take into account the benefits of inorganic perovskites’ inherent photostability and ensure the achievement of high efficiency of a single-cell structure and cutting-edge approaches like replacing the materials.

PSCs comprised of a CsPb._625_Zn._375_IBr_2_ absorber layer are promising because of their opt electrical properties^31,32^. Based on our knowledge, CsPb_0,625_Zn_0.375_IBr_2_ absorber material has not yet been explored experimentally. However, similar halide perovskites, such as CsSn_x_Ge_1−x_IyBr_3 − y_, CsPb_x_Sn_1−x_IyBr_3 − y_ have been successfully synthesized and studied in experimental setups^33–35^. Prior studies have identified several significant challenges during fabrication, including maintaining stability, preventing defects, achieving homogeneous morphology, and controlling stoichiometry^36^. According to those investigations, maximizing the characteristics and device performance of these above-mentioned perovskites requires controlling morphological defects, such as surfaces or heterointerfaces, intragrain defects, and grain boundaries^37,38^. Achieving a high-quality perovskite thin film with characteristics such as dense, uniform, pinhole-free layers, large grain sizes, and low grain boundary density has proven beneficial for device performance. Common fabrication methods such as one-step spin-coating and two-step deposition also have been employed to synthesize these perovskite thin films for achieving high-quality uniform layers. To enhance stability especially additive engineering has been applied in similar perovskite films, which may also be applicable to CsPb_0,625_Zn_0.375_IBr_2_^39,40^.

In addition, the determination of adequate ETL and HTL amalgamation can significantly contribute towards increasing the PCE of these emerging devices^41–43^. Moreover, the thicknesses of the ETL HTL, along with their interface and phase-matching properties, profoundly impact solar metrics including PCE, fill factor (FF), short-circuit current density (J_SC_), and open-circuit voltage (V_OC_)^41–45^.

Specifically, the ETL is an indispensable aspect of a PSC because it extracts electrons from the perovskite absorber and inhibits holes in it. To further boost the PCE of PSC, the scientific community has been meticulously tracking the introduction of new materials through the previously indicated pathway, especially in ETL. TiO_2_, ZnO, and SnO_2_ are examined extensively for PSCs^46–49^. New ETLs are still one of the major concerns in the research community. Recently, Liu et al. achieved 11.2% efficiency with all-low-temperature processed PSCs using CdS as ETL. Peng et al. reported an even higher efficiency of 15%^50^. Tin Sulfide (SnS_2_) is a cost-effective metal sulfide with unique chemical and structural properties, making tunable bandgap ideal for PSC^51^. After studying Mg-doped ZnO (MZO) and the impact of Mg concentration on their optical characteristics and structure, it was determined that Mg-doped ZnO films could be appropriate for PSCs^52^. Zinc Selenide (ZnSe) has been devoted as an ETL in PSCs due to its excellent electron mobility and 2.8 eV straight bandgap. It might function as an n-type collecting layer for reliable and productive commercial PSCs^53^. PSCs with as-deposited [6, 6]-phenyl-C60-butyric acid methyl ester (PC_60_BM) layer displayed no photocurrent hysteresis, even without thermal treatment^54^. So, in comparative analysis, these five ETLs performed superbly in PSCs.

Conversely, though, the HTL impacts solar device manufacturing costs, stability, and efficacy. Materials that are both organic and inorganic are utilized to find the more efficient, stable, and low-cost HTLs^55–57^. However, recent research shows that the superior band alignment, affordability, and stability of inorganic and small molecule HTLs contribute to improved solar cell performance^58–63^. Different organic and inorganic HTLs, Zinc Phosphide (Zn_3_P_2_), Poly[bis(4-phenyl)(2,4,6-trimethylphenyl)amine (PTAA), Molybdenum disulfide (MoS_2_), Molybdenum trioxide (MoO_3_), Poly[2-methoxy-5-(2’-ethylhexyloxy)-1,4-phenylene vinylene] (MEH-PPV), Gallium arsenide (GaAs), Copper aluminum oxide (CuAlO_2_), Copper Telluride (Cu_2_Te), Zinc Telluride (ZnTe), Molybdenum Telluride (MoTe_2_), Copper zinc tin sulfide (CZTS), copper zinc tin selenide (CZTSe), copper manganese tin sulfide (CMTS), and copper-nickel tin sulfide (CNTS) will be analyzed in PSCs because of its tunable band gap.

In this work, we outline the layout and execution of a CsPb._625_Zn._375_IBr_2_ -based perovskite-based solar cell using a unique device architecture: FTO/ETL/CsPb._625_Zn._375_IBr_2_/HTL/Au. This work considers HTLs Zn_3_P_2_, PTAA, MoS_2,_ MoO_3,_ MEH-PPV, GaAs, CuAlO_2_, Cu_2_Te, ZnTe, MoTe_2_, CMTS, CNTS, CZTS, CZTSe as HTLs, CdS, SnS_2_, ZnSe, PC_60_BM as ETLs. To leverage optimal cell output, we offer detailed investigations of the effects of the degree of doping and ETL/HTL layer thickness, perovskite interface layers, electron/hole transport layer separation, absorber layer thickness, and perovskite defect density on PV parameters using the SCAPS-1D in this work^64^. Furthermore, the ramifications of recombination rates, J-V, QE, operating temperature, and series and shunt resistance were assessed in PV performance generation. Finally, a comparison with earlier research was done using the discovered solar cell characteristics. These results imply that our method of device optimization offers a special set of abilities to PSC research that may be used in a real-world device fabrication process in the lab, saving the researchers money and time.

## Device modeling and device structure

### Device modeling

SCAPS-1D has been employed to simulate the device characteristics of perovskite-based solar cells. With SCAPS-1D, a strong correlation between simulation and experimental data could be demonstrated^65^. The SCAPS-1D software for simulating optoelectronic devices was developed by the Department of Electronics and Information Systems (ELIS) at the University of Gent^64^. SCAPS is frequently employed in simulations of optoelectronic devices, notably for solar energy device research since it is capable of resolving the Poisson equations. (Eq. 1) and continuity equations (Eqs. 2–3) to estimate PV device output^64^.

1 $$\:\frac{\partial\:}{\partial\:x}\left({\upepsilon\:}\frac{\partial\:\psi\:}{\partial\:x}\right)=\:-\text{q}\left[\text{p}-\text{n}\:+\:{N}_{D}^{+}-{N}_{A}^{-}+\:\frac{{\rho\:}_{def}}{q}\right]$$

2 $$\:\frac{\partial\:n}{\partial\:x}=\:-\frac{\partial\:{J}_{n}}{\partial\:x}+G-{R}_{n}$$

3 $$\:\frac{\partial\:p}{\partial\:x}=\:-\frac{\partial\:{J}_{p}}{\partial\:x}+G-{R}_{p}\:$$

Where J_n_ and J_p_ are considered electron and hole concentrations respectively. Which are described in (Eqs. 4–5).

4 $$\:{J}_{n}=\:-\frac{{\mu\:}_{n}n}{q}\:\frac{\partial\:{E}_{Fn}}{\partial\:x}$$

5 $$\:{J}_{p}=\:+\frac{{\mu\:}_{p}p}{q}\:\frac{\partial\:{E}_{Fp}}{\partial\:x}\:$$

SCAPS may accept seven distinct material layers and front and back contact layers. Furthermore, SCAPS contextualizes and provides an impeccable venue for this research due to its user-friendly options, which include various defect energy distributions (Gauss, Uniform, tail, single level, or combination), intricate defect shapes (interface or bulk defect), and defect charge types (idealization, monovalent, divalent, and multivalent)^64^. Photovoltaic characteristics (efficiency, J_SC_, FF, and V_OC_) with defect density (Eqs. 6–10) to forecast PSCs characteristics^2^.

6 $$\:L=\:\sqrt{D\tau\:}$$

7 $$\:D=\:\frac{\mu\:{K}_{B}T}{q}$$

8 $$\:\tau\:=\frac{1}{{N}_{T}\delta\:{\vartheta\:}_{th}}$$

9 $$\:FF=\frac{{v}_{oc}-\text{ln}({v}_{oc}+0.72)}{{v}_{oc}}$$

10 $$\:{V}_{oc}=\frac{nKT}{q}\:{v}_{oc}$$

This study focuses on device optimization based on the PV parameters of solar cell devices. SCAPS-1D is a powerful simulator that can model the electrical characteristics of solar cells^66–69^, including current-voltage (I-V) curves, capacitance-voltage (C-V) relationships, capacitance-frequency (C-f) responses, quantum efficiency profiles, and so on. Such electrical modeling plays a crucial role in determining solar cell device performance, enabling the optimization of parameters like layer thickness, doping density, and defect density. However, it is important to acknowledge that optical simulations also play a critical role in optimizing the design of solar cells as supported by previous studies^70,71^. Optical modeling not only enables more accurate calculations of layer thicknesses but also provides valuable insights into material selection for enhancing light absorption and minimizing reflection. Three macroscopic parameters including photon absorption, the energy band gap of the material, and device resistance—are crucial for determining power-conversion efficiency^72–75^. Additionally, in thin-film solar cells, optical interference effects significantly influence light absorption, underscoring the necessity of optical modeling for improving absorption efficiency. However, optical modeling was omitted in this study as our primary objective was to establish a solid understanding of the device’s electrical characteristics.

### Device structure

**Fig. 1:**
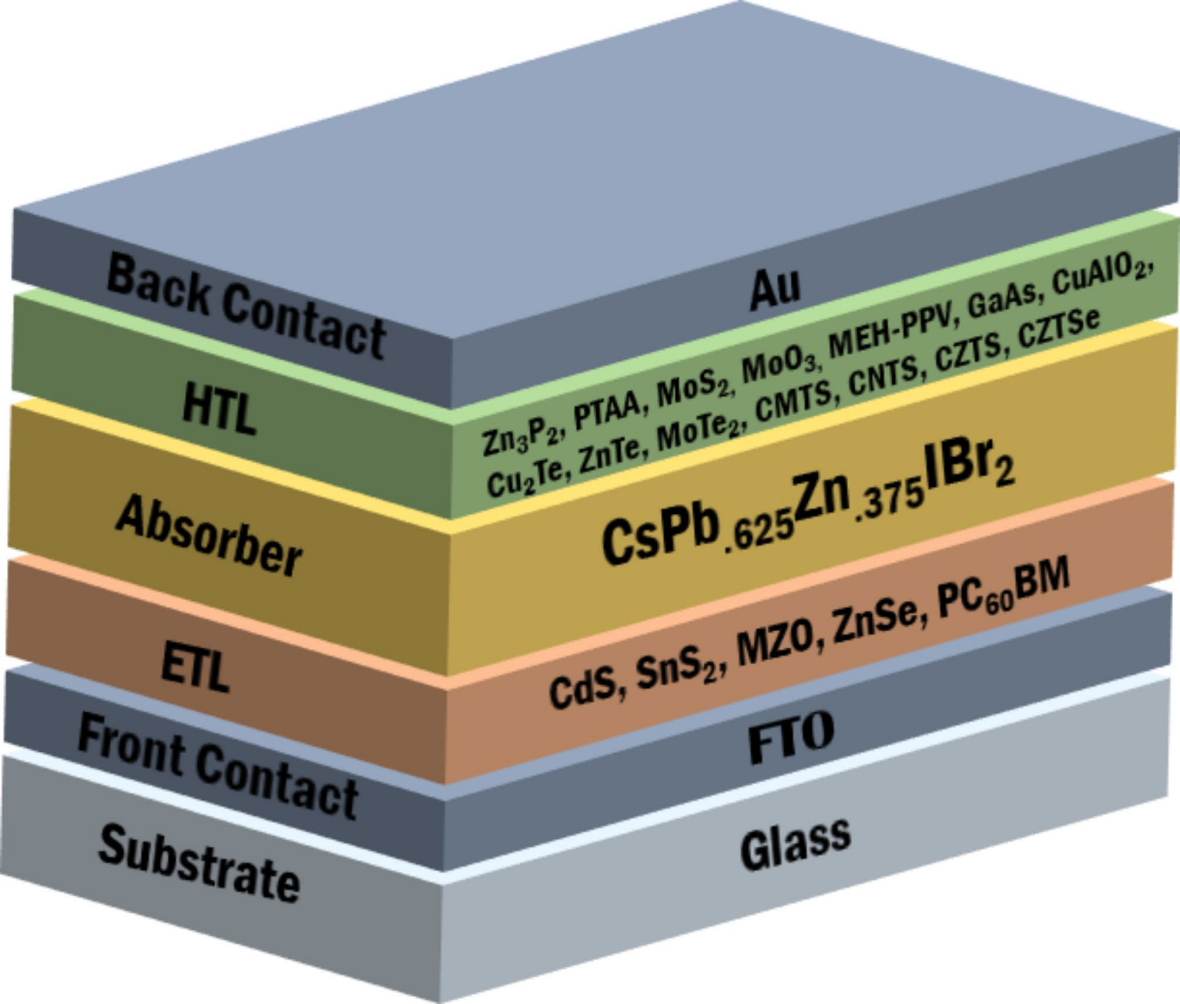
PSC structure based on CsPb._625_Zn._375_IBr_2_ absorber.

An n-i-p planar heterojunction structure consisting of the ETL, HTL CsPb._625_Zn._375_IBr_2_ absorber, transparent Fluorine doped tin oxide (FTO), and gold (Au) back contact was simulated on SCAPS-1D for this investigation, as shown in Fig. 1. In every device structure, the absorber layer CsPb._625_Zn._375_IBr_2_ lies in the space between the HTL and the ETL. The p-region is represented by the HTL, the i-region by the CsPb._625_Zn._375_IBr_2_ absorber, and the n-region by the ETL. When exposed to light, the absorber layer of the solar cell forms electron-hole pairs, with the electrons and holes moving in the directions of the n- and p-layers, respectively. Electrons and holes can migrate and separate because of the electrical field that exists beneath the two layers.

The study examines the efficacy and efficiency of ten HTL and four ETL-based optimized PSC structures. As used in the SCAPS-1D simulation in this work, Tables 1 and 2 displays the optoelectronic properties of the FTO, ETLs (CdS, SnS_2_, ZnSe, PC_60_BM), absorber layer (CsPb._625_Zn._375_IBr_2_), and HTLs (Zn_3_P_2_, PTAA, MoS_2,_ MoO_3,_ MEH-PPV, GaAs, CuAlO_2_, Cu_2_Te, ZnTe, MoTe_2_, CMTS, CNTS, CZTS, CZTSe). With 4.4 eV of work function, an FTO is utilized and the thickness of FTO is assumed to be 200 nm. Device performance is optimized by varying the thickness of the absorber, HTL, and ETL layers across a wide range. The temperature at which the simulation runs is 300 K under a single sun’s radiation (100 mW/cm^2^, AM1.5G).

**Table 1:** Input parameters of the FTO, absorber, and ETL in this study.

Material property	FTO	SnS_2_	ZnSe	CdS	PC_60_BM	CsPb_0.625_Zn_0.375_IBr_2_ (absorber)
Thickness (nm)	200	150	70	50	50	50–500 nm
Bandgap, *Eg* (eV)	3.5	1.85	2.81	2.4	1.8	1.05
Electron affinity, Χ (eV)	4.00	4.26	4.09	4.18	4.2	4.27
Relative dielectric permittivity, εr	9.00	17.7	8.6	10	4	6
Conduction band effective density of states *N*_*C*_ (1/cm^3^)	2.2$\:\times\:$10^18^	7.32$\:\times\:$10^18^	2.2$\:\times\:$10^18^	2.2$\:\times\:$10^18^	1$\:\times\:$10^21^	1 × 10^19^
Valence band effective density of states *N*_*V*_ (1/cm^3^)	1.8$\:\times\:$10^19^	1$\:\times\:$10^19^	1.8$\:\times\:$10^18^	1$\:0.9\times\:$10^19^	$\:2\times\:$10^20^	1 × 10^19^
Electron thermal velocity (cm s^− 1^)	10^7^	10^7^	10^7^	10^7^	10^7^	10^7^
Hole thermal velocity (cm s^− 1^)	10^7^	10^7^	10^7^	10^7^	10^7^	10^7^
Electron mobility, µn (cm^2^/Vs)	20	50	4$\:\times\:$10^2^	100	0.1	25
Hole mobility, µh (cm^2^/Vs)	10	25	1.1$\:\times\:$10^2^	25	0.1	25
Donor density, *N*_*D*_ (1/cm3)	10^18^	9.85$\:\times\:$10^19^	1$\:\times\:$10^18^	1$\:\times\:$10^18^	1$\:\times\:$10^17^	1 × 10^15^
Acceptor density, *N*_*A*_ (1/cm^3^)	0	0	0	0	0	1 × 10^15^
Total density (cm^− 3^)	10^15^	10^14^	1$\:\times\:$10^15^	1$\:\times\:$10^15^	1$\:\times\:$10^15^	1 × 10^12^
Reference	^76^	^77^	^76^	^78^	^79^	^32^

**Table 2:** Input parameters of HTL in this study.

Material property	PTAA	GaAs	ZnTe	CNTS	MoO_3_	MEH-PPV	CuAlO_2_	MoS_2_	Cu_2_Te	Zn_3_*P*_2_
Thickness (nm)	150	150	250	100	100	50	350	200	250	250
Bandgap, *Eg* (eV)	2.96	1.42	2.25	1.74	3.0	2.1	3.46	1.29	1.18	1.5
Electron affinity, Χ (eV)	2.3	4.07	3.73	3.87	2.3	2.8	2.5	4.2	4.2	4.2
Relative dielectric permittivity, εr	9	12.9	7.3	9	18	3	60	3	10	7.11
Conduction band effective density of states *N*_*C*_ (1/cm^3^)	2.0 × 10^21^	2.2 × 10^18^	2.2$\:\times\:$10^18^	2.2$\:\times\:$10^18^	1$\:\times\:$10^19^	2.5$\:\times\:$10^19^	2.2$\:\times\:$10^18^	2.2$\:\times\:$10^18^	7.8$\:\times\:$10^17^	2.2$\:\times\:$10^18^
Valence band effective density of states *N*_*V*_ (1/cm^3^)	2.0 × 10^21^	1.8$\:\times\:$10^19^	1.8$\:\times\:$10^19^	1.8$\:\times\:$10^19^	2.2$\:\times\:$10^18^	2.5$\:\times\:$10^19^	1.8$\:\times\:$10^19^	1.9$\:\times\:$10^19^	1.6$\:\times\:$10^19^	1.8$\:\times\:$10^19^
Electron thermal velocity (cm s^− 1^)	10^7^	10^7^	10^7^	10^7^	10^7^	10^7^	10^7^	10^7^	10^7^	10^7^
Hole thermal velocity (cm s^− 1^)	10^7^	10^7^	10^7^	10^7^	10^7^	10^7^	10^7^	10^7^	10^7^	10^7^
Electron mobility, µn (cm^2^/Vs)	1	8500	300	11	210	0.5$\:\times\:$10^−4^	2	100	500	1
Hole mobility, µh (cm^2^/Vs)	40	400	100	11	210	0.5$\:\times\:$10^−5^	8.6	150	100	10
Donor density, *N*_*D*_ (1/cm3)	0	0			0	0	0	0	0	0
Acceptor density, *N*_*A*_ (1/cm^3^)	1$\:\times\:$10^18^	1$\:\times\:$10^11^	1.0$\:\times\:$10^16^	1.0$\:\times\:$10^19^	1$\:\times\:$10^18^	1$\:\times\:$10^15^	3$\:\times\:$10^18^	1$\:\times\:$10^17^	1.0$\:\times\:$10^21^	1.0$\:\times\:$10^19^
Total density (cm^− 3^)	Acceptor 1$\:\times\:$10^15^	1$\:\times\:$10^14^	1$\:\times\:$10^14^	1$\:\times\:$10^14^	1$\:\times\:$10^15^	1$\:\times\:$10^15^	1$\:\times\:$10^15^	1$\:\times\:$10^14^	1$\:\times\:$10^14^	1.0$\:\times\:$10^14^
Reference	^77^	^80^	^81^	^82^	^83^	^84^	^76^	^85^	^81^	^86^

## Result and discussion

### Impact of different HTL materials on PSC performance

HTL is one of the important factors for achieving high-performance PSCs. Their main purpose is to minimize charge recombination by effectively extracting and transporting photo-generated holes from the perovskite material to the back electrode. For a material to be considered an effective hole transport material, it must fulfill several important requirements. The HTL and the perovskite material should have a favorable energy level alignment for effective hole extraction. It should also have outstanding conductivity and high hole mobility to facilitate efficient charge transfer^87^. Inorganic and organic materials are used to find the best HTL for CsPb._625_Zn._375_IBr_2_-based PSC. In the beginning, we selected SnS_2_ as ETL and designed the device FTO/SnS_2_/CsPb._625_Zn._375_IBr_2_/HTL/Au. We varied HTLs (Zn_3_P_2_, PTAA, MoS_2,_ MoO_3,_ MEH-PPV, GaAs, CuAlO_2_, Cu_2_Te, ZnTe, CNTS) to get the best HTL material for our device. In PSCs, characteristics of HTL like bandgap, band offset, and carrier mobility are considered prevalent roles for getting high efficiency. Valence band offset. Among all of them, valence band offset (VBO) is particularly important and can be measured from the contrast between the absorber’s valence band and HTL and described in Eqs. 11–12 ^88^.

11 $$\:{\chi\:}_{absorber}+\:{{E}_{g}}_{absorber}\ge\:\:{\chi\:}_{HTL}+\:{{E}_{g}}_{HTL}\:\:\:;\:Negative\:VBO\:$$

12 $$\:{\chi\:}_{absorber}+\:{{E}_{g}}_{absorber}\:\le\:\:{\chi\:}_{HTL}+\:{{E}_{g}}_{HTL}\:\:\:;\:Positive\:VBO\:$$

**Fig. 2:**
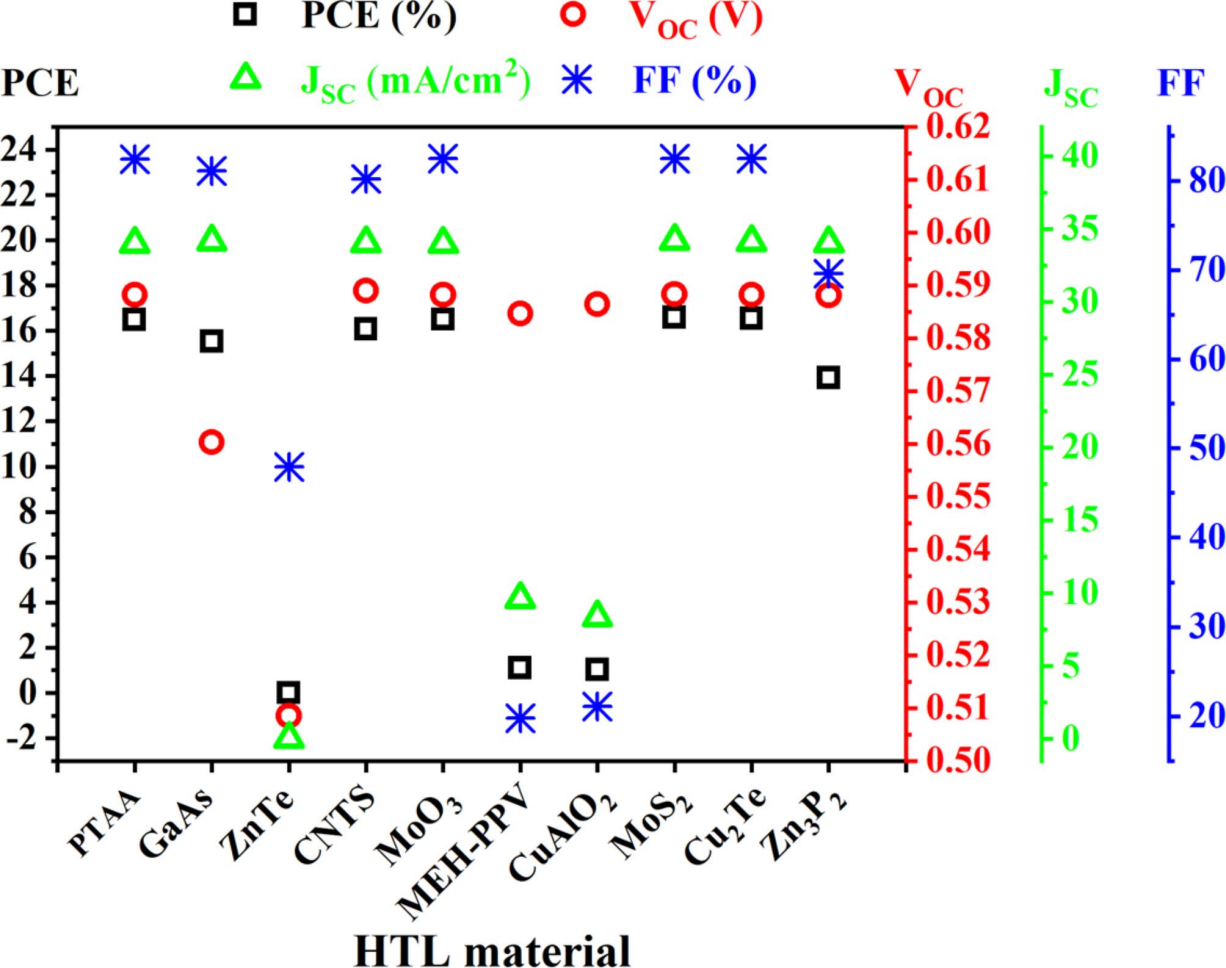
Impact of different HTL materials on device performance.

When VBO becomes negative, a cliff forms between the interface of the absorber and HTL. This cliff does not create a problem with to flow hole toward the electrode. Nevertheless, carrier recombination’s activation energy diminishes and increases the recombination rate^88^. When VBO becomes positive a spike appears at the HTL and absorber interfaces, blocking hole flow to the electrode but, the carrier recombination will probably be reduced. The performance of the cell is negatively impacted if VBO increases because it creates an energy barrier that prevents photogenerated holes from accessing the electrode^88^. However, highly negative VBO raises the energy barrier at the interface, which results in inefficient charge transfer and decreases the device’s performance. Due to the increased barrier, surface recombination is encouraged, which lowers the open-circuit voltage and overall device efficiency^89,90^.

Following the evaluation of various HTL materials on PSCs performance, the device incorporating MoS_2_ HTL exhibited the highest PCE, as shown in Fig. 2. This superior performance prompted the selection of MoS_2_ as the HTL for further numerical analysis. A positive VBO (+ 0.15 eV) creates a spike that reduces the carrier recombination and gets a higher efficiency (16.59%) compared to the other HTL.

### Band diagram and ETL dependent device performance

**Fig. 3:**
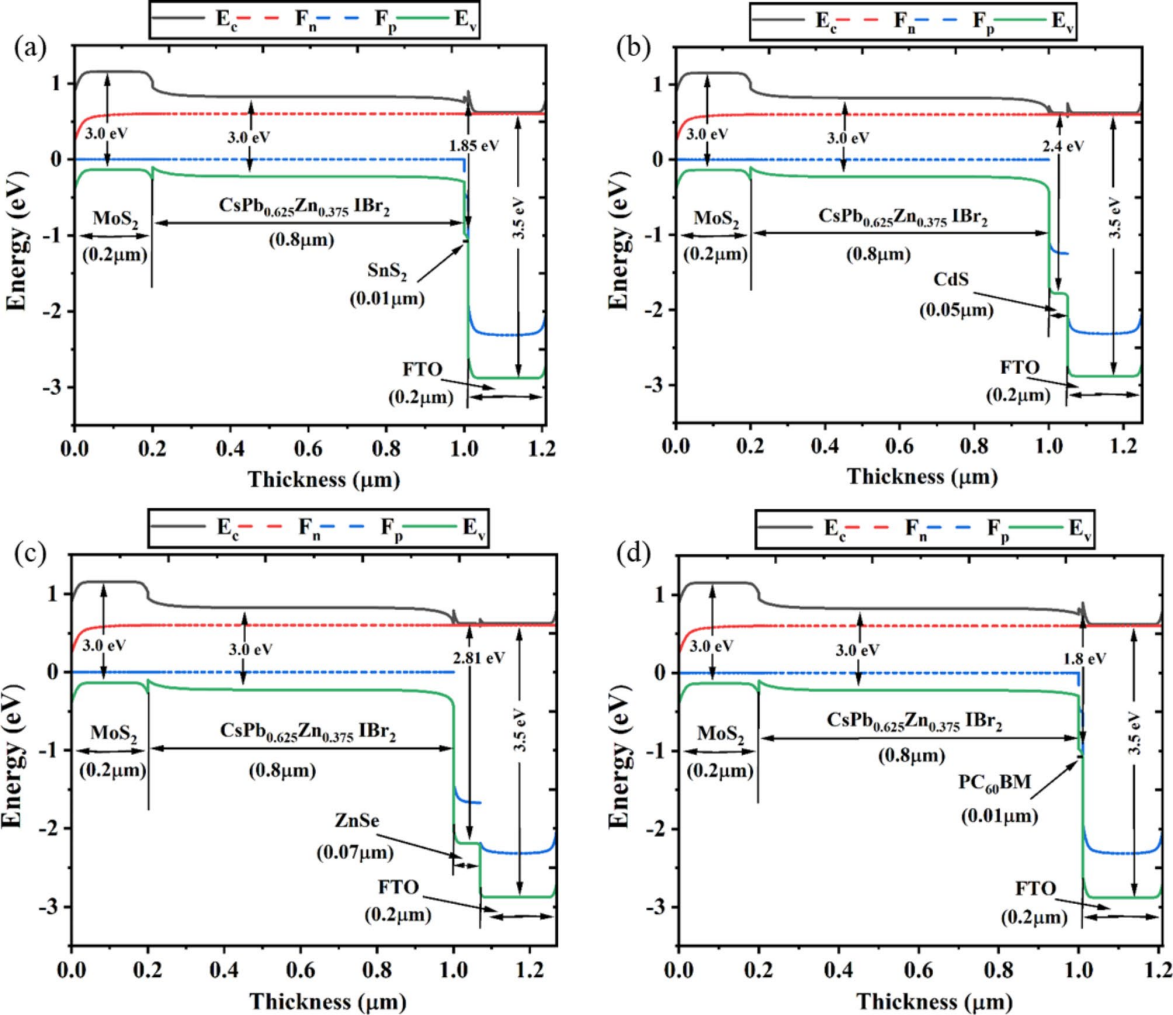
Energy band diagram of PSCs with distinct ETLs as (**a**) SnS_2_, (**b**) CdS, (**c**) ZnSe, (**d**) PC_60_BM.

The band alignment of the CsPb._625_Zn._375_IBr_2_-based PSC device setup is shown in Fig. 3, where the MoS_2_ HTL and SnS_2_, PC_60_BM, ZnSe, and CdS are utilized as the ETLs. The absorbing layer of each ETL and HTL is used by the valence band offset to be impacted by the energy band diagram and conduction band offset, respectively. The energy level alignment impacts the performance of PSCs. In the PSCs, the flow of electrons is introduced into the matching ETL conduction band while holes are concurrently transported to the HTL^91^. Subsequently, the corresponding Au and FTO are where holes and electrons are gathered, respectively.

The efficiency of PSCs is profoundly impacted by the energy-level alignment. Photogenerated electrons are simultaneously transported to the HTL by holes and injected into the ETL conduction band in PSCs. Subsequently, the front (FTO) and rear (Au) contact metals accumulate electrons and holes, respectively. The ionization energy of HTL must be less than CsPb._625_Zn._375_IBr_2_, and ETL’s electron affinity must be greater than the CsPb._625_Zn._375_IBr_2_, to extract the holes at the CsPb._625_Zn._375_IBr_2_/HTL interface. Key performance measures of the device, including the J_SC_, V_OC_, FF, and PCE, are also profoundly altered by the mismatch in energy bands at the ETL/CsPb._625_Zn._375_IBr_2_ and CsPb._625_Zn._375_IBr_2_/HTL interface^91^. The Fermi levels adjacent to the band of valence differ from one another, as Fig. 3 illustrates, but they are in proximity to the conduction band and progressively penetrate it in all four scenarios. Figure 3 displays the energy band map for four distinct solar cell architectures: SnS_2_, CdS, ZnSe, and PC_60_BM as ETL. The HTL (MoS_2_) and the built-in potential of the absorber interface are important factors in influencing the J_SC_ and V_OC_ of PSCs. Electron transport, or the movement of negative charge carriers from the perovskite layer to the electrode, is the function of the ETL. The J_SC_ and V_OC_ as well as the overall performance of the solar cell can be greatly impacted by the kind and quality of the ETL^91^. An efficient ETL can boost the current density by reducing hole and electron recombination at the contact between the ETL and the perovskite layer^91^. However, to understand the band alignment between the absorber and different ETLs, it is crucial to analyze the Conduction Band Offset (CBO), which can be calculated as $\:\text{C}\text{B}\text{O}={{\upchi\:}}_{\text{a}\text{b}\text{s}}-{{\upchi\:}}_{\text{E}\text{T}\text{L}}$^92^. A positive CBO results in a spike-like band alignment, where the absorber’s conduction band is lower than that of the ETL. In contrast, a negative CBO leads to a cliff-like band alignment, where the absorber’s conduction band is higher than the ETL’s. As shown in Fig. 3, most ETL devices exhibit a negative CBO, indicating smoother electron extraction from the absorber to ETL^93,94^. Due to variations of work function in the ETL and absorber, an almost entirely depleted absorber layer forms in the CsPb._625_Zn._375_IBr_2_-based device, as seen in Fig. 3. The passage of charge carriers within the PSCs was influenced by the internal electric field created by this depleted absorber layer. Higher short circuit current (J_SC_) is the outcome of a fully depleted absorber layer; however, a lower V_OC_ value is caused by an inadequate offset between the absorber/HTL and the quasi-fermi level from the ETL/absorber^91^.

### Optimization of absorber, ETL, and HTL thickness

#### Impact of absorber thickness

**Fig. 4:**
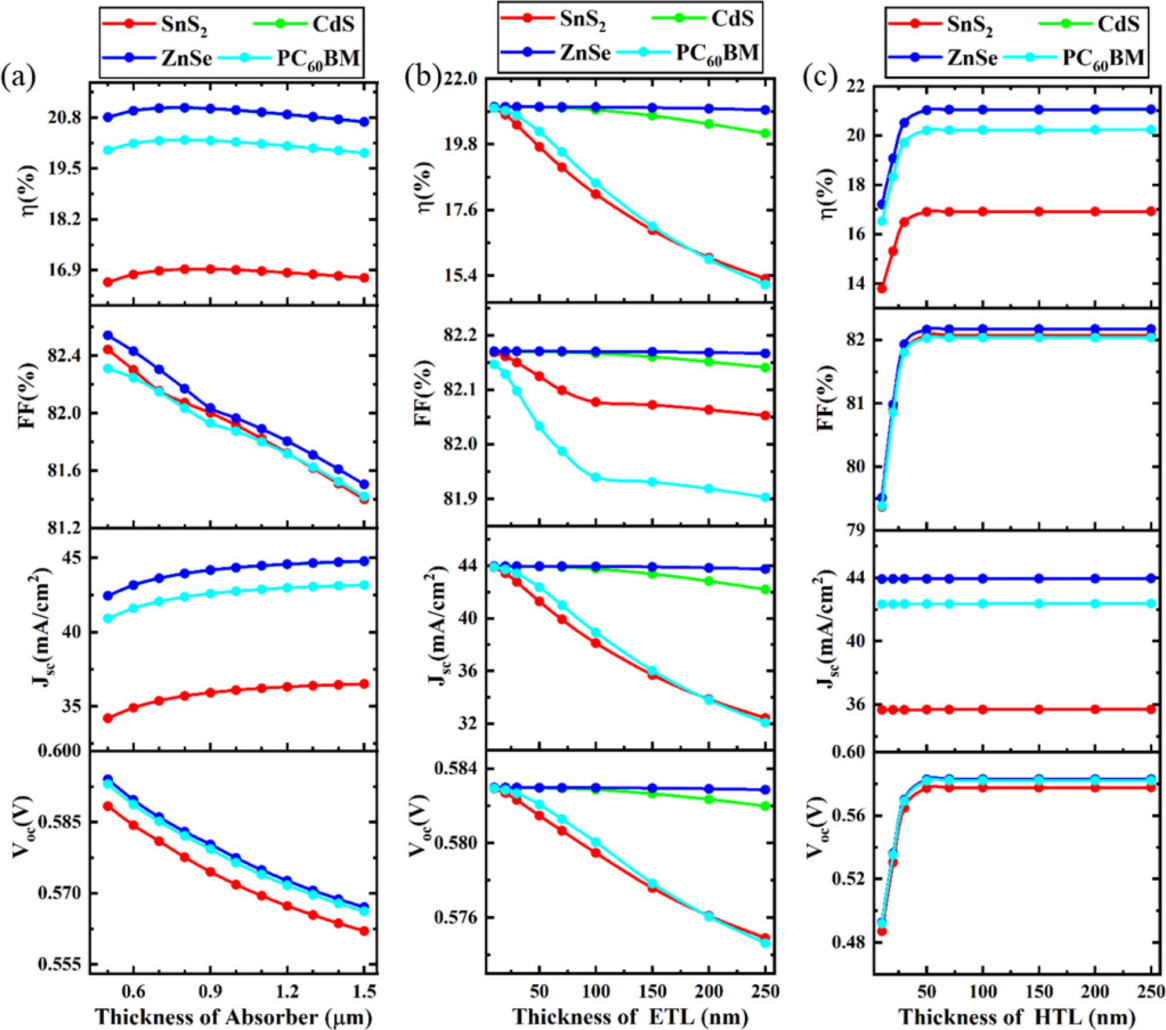
Impact of the absorber, ETL, and HTL thickness on device performance.

The absorber layer’s thickness has a major impact on how well PSC functions. Figure 4 FTO/ETL/CsPb._625_Zn._375_IBr_2_/MoS_2_/Au PSCs are analyzed with varying absorber, ETL, and HTL thicknesses. Figure 4a illustrates the impact of absorber thickness by varying the absorber’s thickness ranges from 0.5 μm to 1.5 μm. V_OC_ changes ridiculously small when the thickness is varied. When the thickness is 0.5 μm V_OC_ is almost 0.593 V and after increasing the thickness V_OC_ decreases and reaches 0.562 V. This agrees well with values that have been previously published^7,95^. According to Fig. 4a, J_SC_ rises as absorber thickness increases. The photogeneration of excitons and light absorption are responsible for this increase in J_SC_. Due to its comparatively poor absorption, a thin absorber layer produces few electron-hole pairs at a large wavelength range. As the perovskite absorber’s thickness expands, the long wavelength absorption rises, improving exciton pair formation. In all four different ETL configurations, J_SC_ is increasing with thickness increments^7,95^. However, in solar cell configurations, increasing thickness adds to the series resistance, resulting in substantial carrier recombination losses and decreasing FF^96^. Figure 4 shows that PCE and J_SC_ improve up to 700 nm as the absorber layer thickness grows from 0.2 μm to 1 μm, indicating higher creation of electron-hole pairs^97,98^. However, an absorber thickness of 0.8 μm has been maintained for further numerical analysis of all ETL devices where the highest PCE, 21.05%, was achieved for the ZnSe ETL device, with a V_OC_, J_SC_, and FF of 0.58 V, 43.94 mA/cm^2^, and 82.17%, respectively.

#### Impact of ETL thickness

Extracting electrons from the absorber surface without allowing holes to recombine is the fundamental and main purpose of the ETL. The varying thicknesses of the ETL will alter the visible light transmittance of the film, as demonstrated in experimental settings. Consequently, the thickness of the ETL is especially important for optimizing the solar cell. The fluctuations in ETL thickness from 0.01 μm to 0.25 μm with the output of PSCs may be improved to illustrate the impact of ETL thickness. It is evident from the results shown in Fig. 4b that the PCE reduces as the ETL becomes thicker for SnS_2_, PC_60_BM. This reduction may cause more significant pinholes to form, which reduces the J_SC_. Moreover, a thicker ETL promotes greater electron-hole recombination, increasing resistance and contributing to a decline in PCE^99^. However, PCE is almost constant for ZnSe and CdS ETL devices and this agrees well with values that have been previously published^100^. In this study, an ETL thickness of 0.01 μm has been selected as the optimized value for SnS_2_ and PC_60_BM-based ETL devices, while thicknesses of 0.05 μm and 0.07 μm are optimized for CdS and ZnSe ETL devices, respectively. These values were maintained in further numerical analyses. Among the four ETL configurations, the ZnSe ETL device achieved the highest PCE of 21.05%, with a V_OC_, J_SC_, and FF of 0.58 V, 43.94 mA/cm^2^, and 82.17%, respectively.

#### Impact of HTL thickness

Figure 4c illustrates how performance characteristics in CsPb._625_Zn._375_IBr_2_-based PSCs utilizing ZnSe, PC_60_BM, ZnSe, and CdS as ETLs are affected by varying the thickness of the MoS_2_ HTL. To reduce direct contact between the anode and perovskite and enhance performance, it is imperative to modify the HTL thickness. We exclusively look at MoS_2_ as the HTL in thickness optimizations. This result suggests that while the device’s charge transport properties improved, there may have been an increase in charge recombination, leading to a relatively stable trend in J_SC_. As the HTL thickness increases, carrier collection improves, resulting in a rise in Voc. Consequently, an upward trend in PCE is observed. As the HTL increased in thickness of MoS_2_, Fig. 4c demonstrates that V_OC_, J_SC_, FF, and PCE levels remained unchanged for any of the ETLs. Around 21% PCE for ZnSe for 50 nm thickness of MoS_2_, and this value is consistent with further increments in the thickness of HTL. Similar findings have been reported in previous studies, where an increase in HTL thickness corresponded with enhanced PCE^2,7,101–103^. However, an HTL thickness of 0.02 μm has been maintained for further numerical analysis across all ETL devices. Notably, the ZnSe ETL device again shows the highest PCE of 21.05%, with a V_OC_ of 0.58 V, J_SC_ of 43.94 mA/cm², and FF of 82.17%.

### Optimization of the acceptor, donor, and defect density of the absorber

#### Impact of acceptor density of absorber

**Fig. 5:**
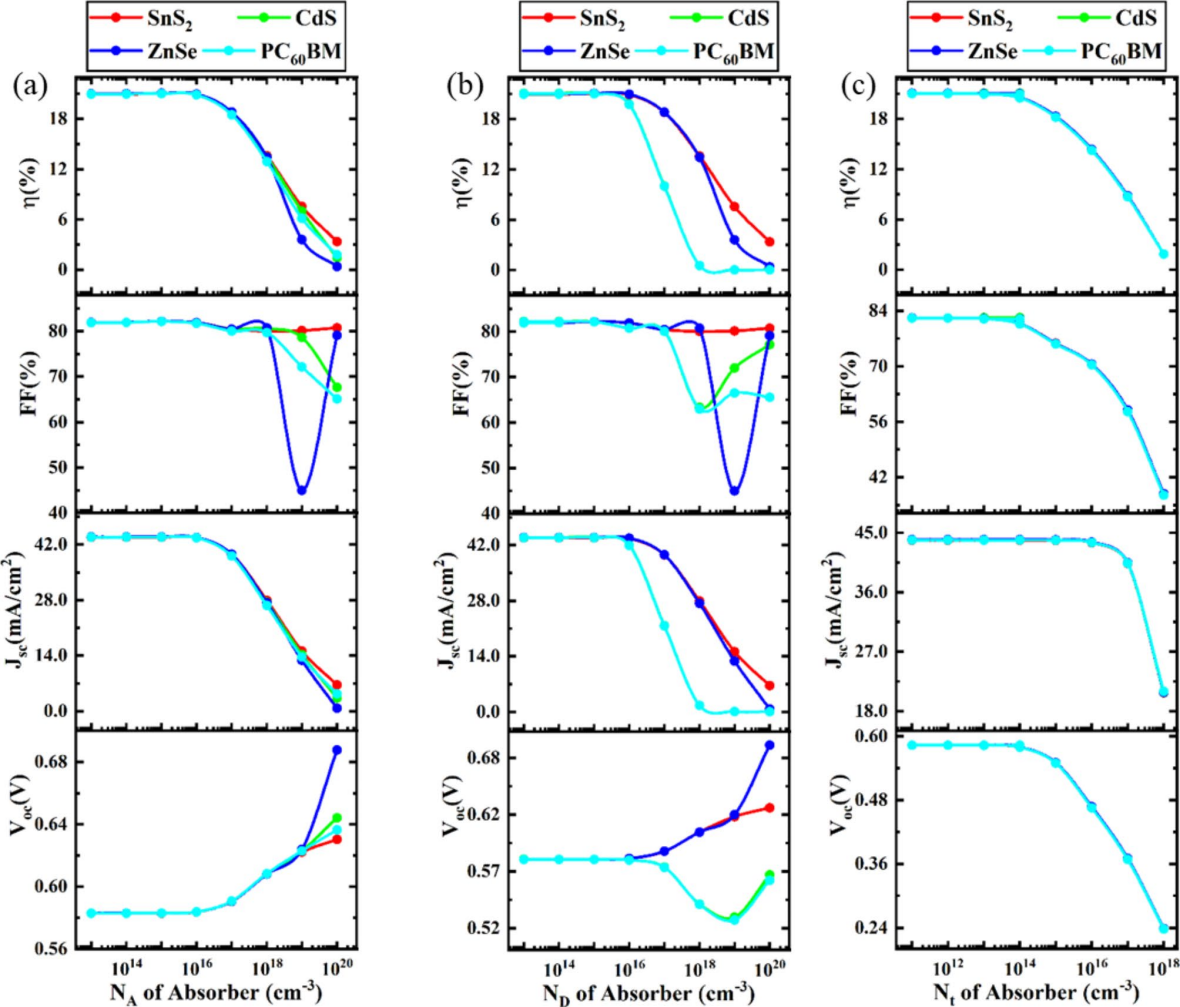
Impact of acceptor, acceptor, and donor density of absorber on PSC performance.

Figure 5a provides descriptive observations on the characteristics of performance following acceptor density (N_A_) change. N_A_ varies in CsPb._625_Zn._375_IBr_2_ absorber from 1 × 10^13^ cm^− 3^ to 1 × 10^20^ cm^− 3^. Four different ETLs (ZnSe, PC_60_BM, ZnSe, and CdS) are analyzed here with MoS_2_ HTL. V_OC_, J_SC_, FF, and PCE are merely constant for N_A_ 1 × 10^13^ cm^− 3^ to 1 × 10^16^ cm^− 3^. So, N_A_ has no influence on cell performance when the value of N_A_ is less than 1 × 10^16^ cm^− 3^. However, when the value of N_A_ crosses the value of 1 × 10^16^ cm^− 3^, V_OC_ starts to increase but J_SC_ and FF start to decrease, and overall device efficiency starts to decrease. Higher doping concentrations may degrade the solar cell’s active layer by introducing unfavorable shunt pathways through the absorber layer, leading to a reduction in the solar cell’s efficiency^104^. The same phenomena are observed in previous works^95,105^. The influence of different types of ETL is limited here. The same characteristics are observed for all types of ETL. However, the optimal absorber acceptor density has been set at 10^5^ cm^− 3^ across all ETL devices, with the ZnSe ETL device demonstrating comparatively higher performance, achieving a PCE of 21.06%, FF of 82.17%, J_SC_ of 43.94 mA/cm^2^, and V_OC_ of 0.58 V.

#### Impact of donor density of absorber

Figure 5b illustrates the device performance if the donor density (N_D_) of absorber CsPb._625_Zn._375_IBr_2_ varies from 1 × 10^13^ cm^− 3^ to 1 × 10^20^ cm^− 3^. To observe the best device configuration for the absorber CsPb._625_Zn._375_IBr_2_, MoS_2_ materials are used for HTL, and four different materials (ZnSe, PC_60_BM, ZnSe, and CdS) are used to get the best performance. Like N_A_, the same performance characteristics are absorbed for N_D_. As the donor density increases, the electric field at the interfaces strengthens, enhancing the charge separation process. However, performance may decline if this stronger field also leads to increased recombination. Additionally, excessive doping can introduce donor defects and increase non-radiative recombination, which may raise the series resistance in PSCs and consequently reduce the PCE. According to Fig. 5b, when the value of N_D_ is higher than 1 × 10^16^ cm^− 3^ the PCE starts to diminish. However, for further numerical analysis, the donor density has been set to 10^5^ cm^− 3^. The ZnSe ETL device demonstrates superior performance among four investigated devices, with a PCE, FF, J_SC_, and V_OC_ of 21.06%, 82.17%, 43.94 mA/cm^2^, and 0.58 V respectively.

#### Impact of defect density of absorber

The absorber defect density (N_t_) has a substantial influence on PSC efficacy. Higher N_t_ levels in the absorber layer cause film degradation and pinhole development, leading to increased recombination and decreased stability and PCE. To determine the optimal defect density, the simulation varied the absorber defect density from 1 × 10^13^ cm^− 3^ to 1 × 10^18^ cm^− 3^.

The plot of photovoltaic parameters (V_OC_, J_SC_, FF, PCE) against perovskite defect density is shown in Fig. 5c. The findings demonstrate that when the CsPb._625_Zn._375_IBr_2_ defect density increases, the PCE of the examined PSC device falls. As the fault density increases, V_OC_ reduces linearly, as seen in Fig. 5c. The short diffusion length of the perovskite materials is the cause of this decrease in V_OC_. The charge carriers are typically trapped by high defect sites in perovskite materials, which shortens their lifespan. Consequently, as this carrier lifetime diminishes, the diffusion length does as well^106^. Nonetheless, J_SC_ exhibits a constant response up to a defect density of around 1 × 10^16^ cm^− 3^, after which it begins to decline as the absorber layer’s defect density increases. This is thus because the J_SC_ is reliant on the pace at which electron-hole pairs are generated. Carrier diffusion is also a factor in J_SC_, and it gets less with increasing defect density. As a result, J_SC_ falls as fault density increases. However, because of the short diffusion length, it dramatically decreases if the defect density surpasses 1 × 10^16^ cm^− 3^^107^. From this observation, it is observed that up to 1 × 10^14^ cm^− 3^ absorber defect density is tolerable beyond this value the performance starts to decrease. The same trends are observed in previously reported articles^105,106^. Based on the PV parameters, a defect density of 10^12^ cm^− 3^ has been selected as the optimal value for further numerical analysis. Under these conditions, the ZnSe ETL device outperforms the other devices, achieving a PCE of 21.05%, FF of 82.17%, J_SC_ of 43.94 mA/cm^2^, and V_OC_ of 0.58 V.

### Optimization of donor and defect density of ETL

#### Impact of donor density of ETL

**Fig. 6:**
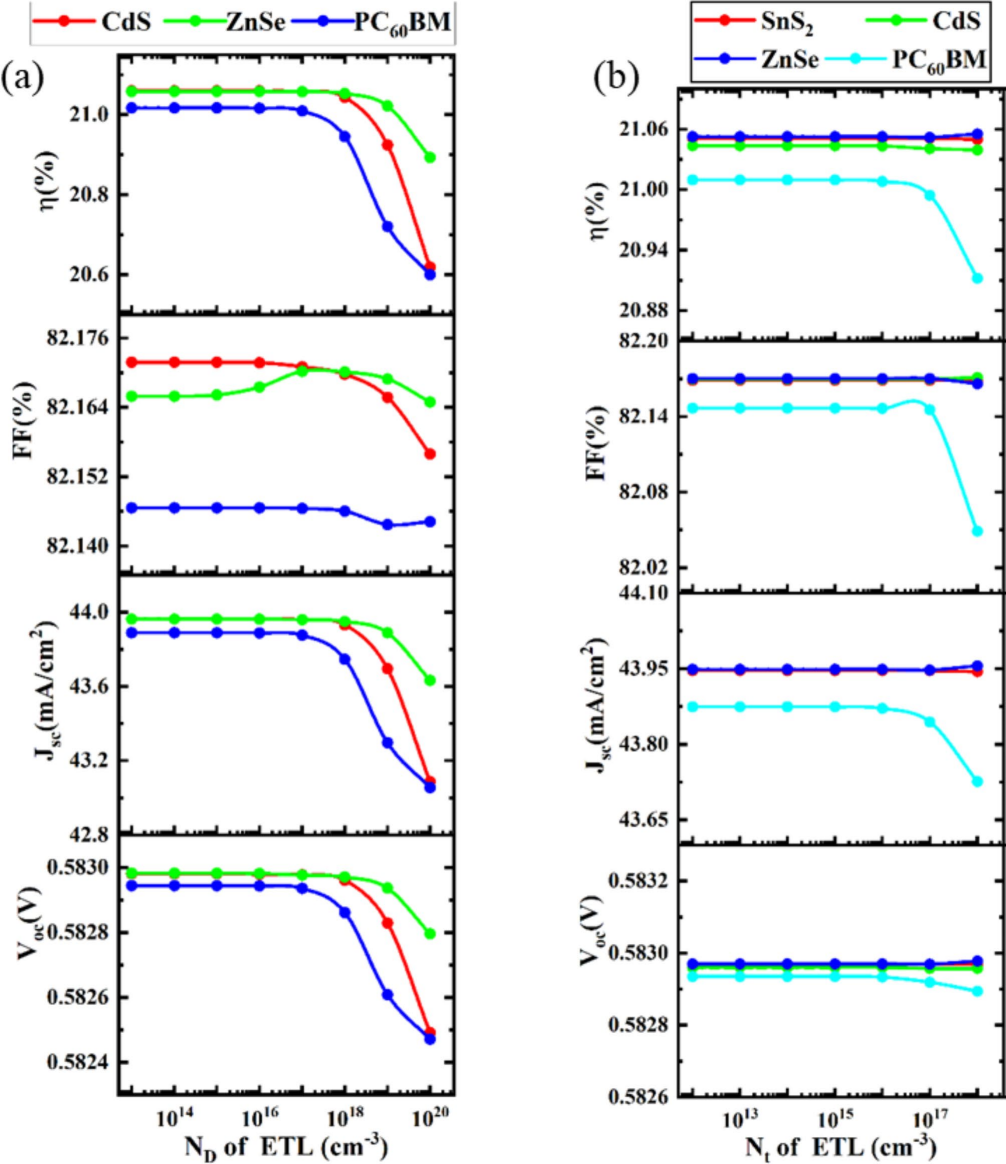
Impact of donor and defect density of ETL on device performance.

The concentration of doping has a major role in separating the charge carriers produced by photolysis. ETL stops hole migration while transferring electrons to the cathode. The electric field that exists at the ETL/absorber contacts and is dependent on the doping density is what separates these charge carriers^108^. This electric field at the interfaces can be strengthened by the doping density concentration in ETL layers. Consequently, when the donor density of ETL grows, the minority carrier concentration is considerably reduced at the interfaces due to a reduction in carrier recombination, which raises excitonic separation^108,109^. To find the best performance, the ETL layer’s doping concentration is changed in this part from 1 × 10^13^ cm^− 3^ to 1 × 10^20^ cm^− 3^. The output parameters of PSC, are also noted. At a resolution of 1 × 10^17^ cm^− 3^, the PSC exhibits optimal performance. It is also evident from Fig. 6a that there is no discernible change in V_OC_ or J_SC_ with the changing of N_D_ of ETL. Keeping the N_D_ in ETL to 1 × 10^17^ cm^− 3^ initiates the subsequent assessment procedure and V_OC_, J_SC_, FF, and PCE are also nearly dramatically decreased. Similar features are noted in earlier published publications^7,110^. However, among all devices, the ZnSe ETL device comparatively shows better performance with a PCE, V_OC_, J_SC_, and FF of 21.05% 0.58 V, 43.94 mA/cm^2^, and 82.17%, respectively.

#### Impact of defect density of ETL

For various ETLs, the values of V_OC_, J_SC_, FF, and PCE are calculated by raising the ETL defect density from 1 × 10^12^ cm^− 3^ to 1 × 10^18^ cm^− 3^. As the N_t_ of the ETL increased, Fig. 6b demonstrated that practically all performance parameters—V_OC_, J_SC_, FF, and PCE—indicated quite constant values for all ETL layers except PC_60_BM. When PC_60_BM is used as ETL all parameters V_OC_, J_SC_, FF, and PCE start to decrease when defect density is higher than 1 × 10^16^ cm^− 3^. PCE significantly decreases due to an increase in recombination pathways and trap states^111^. Therefore, a defect density of 1 × 10^15^ cm^− 3^ can be considered optimal for better performance in all devices. However, the ZnSe ETL device achieves the highest V_OC_ value of 0.58 V, whereas the J_SC_ value of about 48.94 mA/cm^2^. PC_60_BM as the ETL displayed a lower value of J_SC_ (43.87 mA/cm^2^). The same characteristics are observed in previously reported articles^7,95,110^.

### Optimization of acceptor and defect density of HTL

#### Impact of acceptor density of HTL

Corresponding to ETL’s donor density, the consequences of HTL’s acceptor density increase the performance of PCSs. The process of separating the charge carriers generated by sunlight is largely dependent on the acceptor concentration. HTL transfers holes to the anode. These charge carriers are distinguished by an electric field that depends on the acceptor density and is present at the absorber/ HTL contacts^108^. The acceptor density concentration throughout HTL sections can ameliorate the electric field at the interfaces. Consequently, when the acceptor density of HTL increases, fewer carriers recombine at the interfaces, resulting in a significant boost in excitonic separation and a reduction in minority carrier concentration^108,109^.

**Fig. 7:**
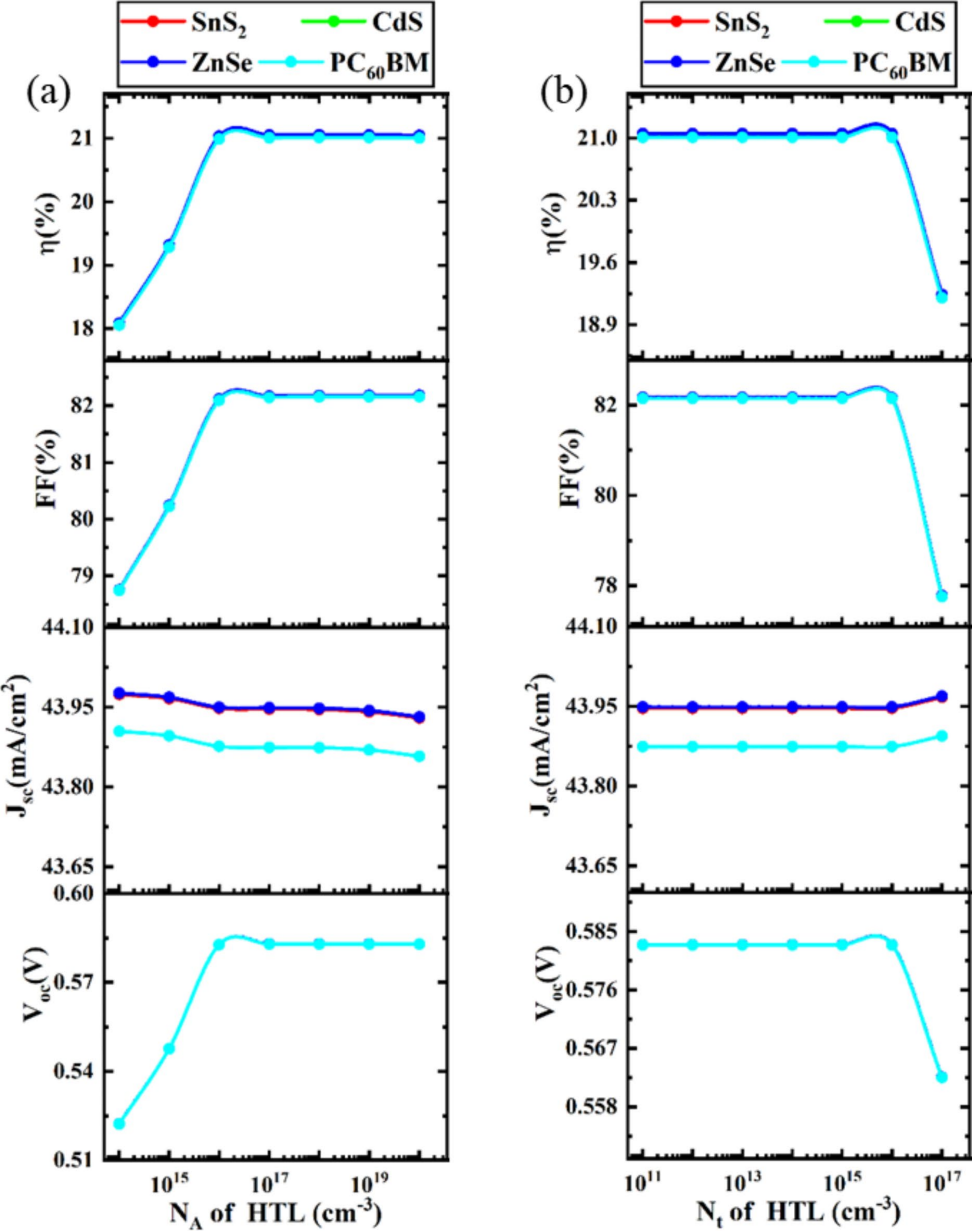
Impact of acceptor and defect density of HTL on device performance.

The change in N_A_ of HTL from 1 × 10^14^ cm^− 3^ to 1 × 10^20^ cm^− 3^, with all other optoelectronic parameters held constant, is shown in Fig. 7a. Up to 1 × 10^16^ cm^− 3^ doping density, all the output parameters, such as V_OC_, FF, and PCE of PSC parameters start increasing while J_SC_ is constant. After increasing acceptor density 1 × 10^16^ cm^− 3^, V_OC_, FF, and PCE remain constant. The optimal value for acceptor doping density is 1 × 10^16^ cm^− 3^ since it exhibits the best PCE. By putting this optimization in place, an additional assessment procedure is carried out. Similar features are noted in earlier published publications^2,7,110^.

#### Impact of defect density of HTL

The values of V_OC_, J_SC_, FF, and PCE are computed for different structures by increasing the HTL defect density from 1 × 10^11^ cm^− 3^ to 1 × 10^17^ cm^− 3^. Figure 7b showed that almost all performance metrics (V_OC_, J_SC_, FF, and PCE) suggested nearly comparable characteristics for all structures when the N_t_ of the HTL rose. V_OC_, FF, and PCE are practically constant up to 1 × 10^16^ cm^− 3^ defect density; beyond that, they begin to drop, whereas J_SC_ is almost constant or all the density. V_OC_, FF, and PCE begin to decline at an increase in acceptor density of 1 × 10^16^ cm^− 3^, although J_SC_ exhibits very little gain. The primary cause of this PCE deterioration is the rapid formation of numerous recombination sites within the HTL and at the interfaces after exceeding 1 × 10^16^ cm^− 3^ of defect density^112^. This increased defect density in the HTL, caused by factors such as dislocations and native defects, leads to the formation of shallow traps. These traps negatively impact the cell’s performance by acting as non-radiative recombination centers^113^. Given that it has the best PCE, 1 × 10^16^ cm^− 3^ is the ideal acceptor doping density. By implementing this optimization, a further evaluation process is completed. Similar characteristics have been reported in past publications^7,95^.

### Effects of series resistance, shunt resistance, and temperature

#### Impact of series resistance on device performance

**Fig. 8:**
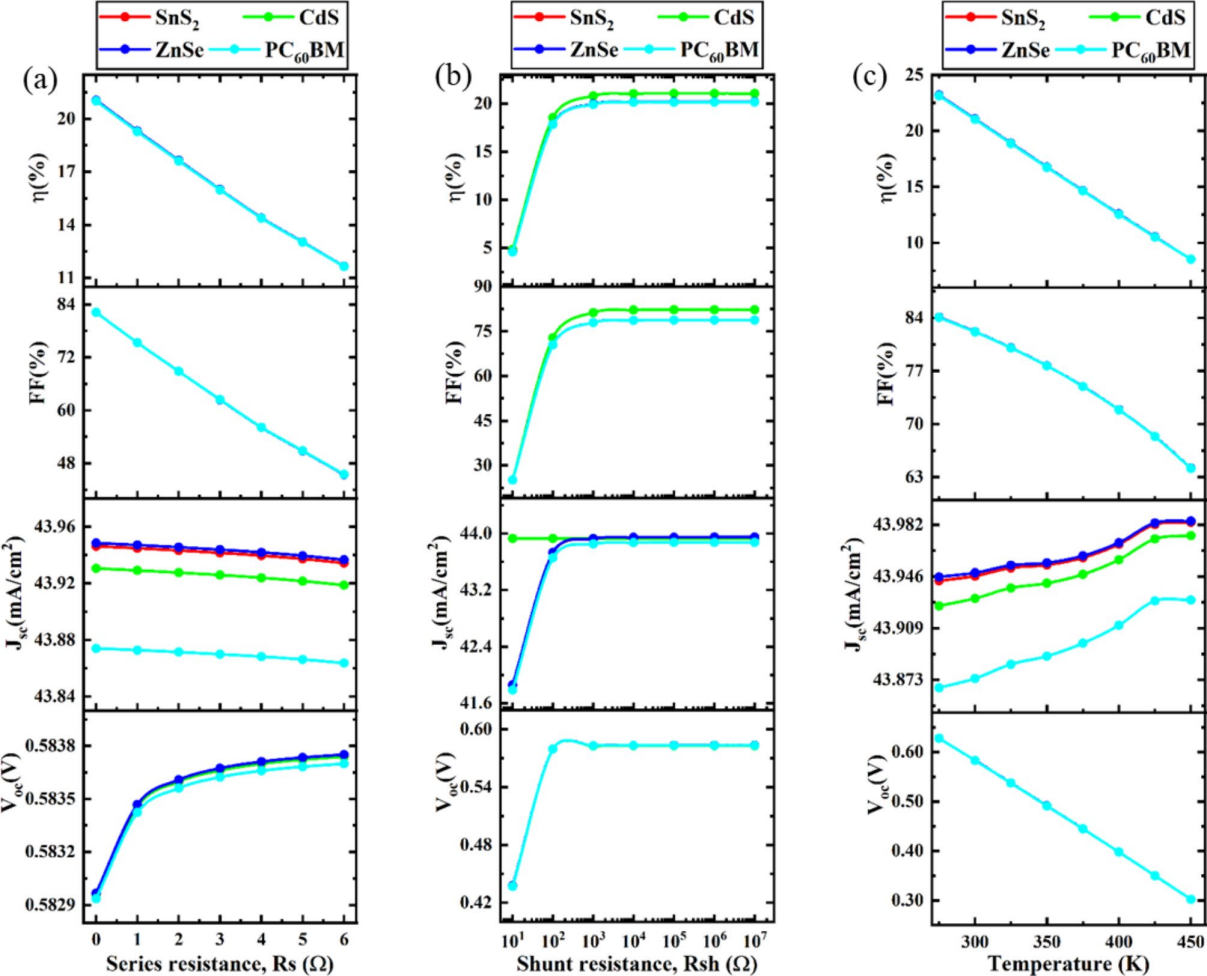
Impact of series resistance, shunt resistance, and temperature on device performance.

The combination of the absorber layer resistance and the ohmic contact resistance of the device is known as series resistance (R_S_). The impact of varying the series resistance for four different ETLs (ZnSe, PC_60_BM, ZnSe, and CdS)-associated structures from 0 Ω-cm^2^ to 6 Ω-cm^2^ is examined, as shown in Fig. 8a. It is shown that J_SC_ and V_OC_ were almost unaffected by increasing R_S_. Nonetheless, the growth in R_S_ has resulted in an enormous downturn in the FF (82–44%) and PCE (21–12%) for all structures. Consequently, throughout the device’s manufacture, R_S_ must be reduced to a minimum to maximize performance and optimize FF. To reduce R_S_, decreasing the thickness of the absorber layer during manufacturing is not a solution because of lowering the thickness of the absorber layer, as doing so causes a noncomplementary absorption^30,110^. Minimizing the resistance to contact between the electrodes and active layer or designing well-interacting donor-acceptor interfaces are two further experimental techniques to reduce R_S_^30,110^. A similar output trend is noted in earlier published publications^114,115^.

#### Impact of shunt resistance on device performance

Materials for absorbers, interface barriers, interlayers that gather charges, and electrodes all contribute to PSCs’ internal resistance. The Shockley equation (Eqs. 13–14) describes how a solar cell’s J-V characteristic responds under ideal one-sun illumination parameters^30,110,114^.

13 $$\:{J}_{SC}=\:{J}_{PH}-\:{J}_{o}\:\left[\text{exp}\left(\frac{{q}_{e}\left(V-J{R}_{S}\right)}{nK{T}_{e}}\right)-1\right]-\:\frac{V-J{R}_{S}}{{R}_{Sh}}$$……….

14 $$\:{V}_{OC}=\:\left(\frac{nK{T}_{e}}{{q}_{e}}\right)\text{ln\:}\left[\frac{{J}_{PH}}{{J}_{O}}\:\left(1-\frac{{V}_{OC}}{{J}_{PH}{R}_{Sh}}\right)\right]$$…………

The equation includes the elementary charge (q_e_), photocurrent density (J_PH_), reverse bias saturation current density (J_o_), series resistance (R_S_), shunt resistance (R_Sh_), diode ideality factor (n), Boltzmann constant (1.38 × 10^23^ JK^− 1^), and ambient temperature (298 K).

Figure 8b illustrates how V_OC_, J_SC_, FF, and PCE values affect R_Sh_ variation, which ranges from 10 Ω-cm^27^ to 10 Ω-cm^2^ for all PSC configurations. As R_Sh_ rose, the PV parameters followed a comparable pattern, rapidly rising from 10 Ω-cm^23^ to 10 Ω-cm^2^. According to Fig. 8b, different ETL-associated structures had the same V_OC_ of 0.58 V. Figure 8b shows that ZnSe, PC_60_BM, ZnSe, and CdS as ETL structures, J_SC_ increased with the fluctuation of R_Sh_ for different ETL-associated structures. After 10^3^ Ω-cm^2^, the J_SC_ for R_Sh_ increased in all PSC structures and remained steady. As for FF, Fig. 8b indicates that all four PSC structures exhibited a rising trend; however, once R_Sh_ variation decreased, CdS, ETL-associated PSC structures, displayed about 80% FF. The remaining four PSC structures linked to ETLs exhibited less than 80% of FF. According to Fig. 8b, nearly the same trend was seen in the PCE cases of CdS, where the structure connected to the ETL has an enhanced PCE of about > 20%. A similar output trend is noted in earlier published publications^114,115^. Thus, this analysis indicates that maximizing shunt resistance is essential for achieving optimal device performance.

#### Impact of temperature on device performance

The power output of a PSC is directly impacted by its operating temperature. Higher operating temperatures are expected to have an impact on the density of states, band gaps, electron and hole mobility, carrier concentrations, and other solar cell properties^2^. Similarly, the operating temperature impacts the proposed cell’s performance parameters. The temperature of this simulation impact is adjusted between 275 k and 450 K. Figure 8C displays the V_OC_ plotted against temperature fluctuation. The graph shows that when the working temperature of the cell rises, the V_OC_ falls, though J_SC_ remains almost constant. The creation of extra interfacial defects, a rise in series resistance, and a short carrier diffusion length may all be employed to clarify the decline in V_OC_ according to the operational temperature^2^. FF and PCE of the raised four models also start decreasing with an increment in temperature. According to our findings, the greatest PCE is produced under the usual test setting of 300 K, and the PCE declines linearly when the temperature escalates. The approximate PCE of the suggested cell at 300 K is 21.05% for the CdS- ETL-based PSC structure. The same phenomenon is noted in earlier published publications^116,117^.

### Effect of absorber layer thickness with absorber acceptor density

**Fig. 9:**
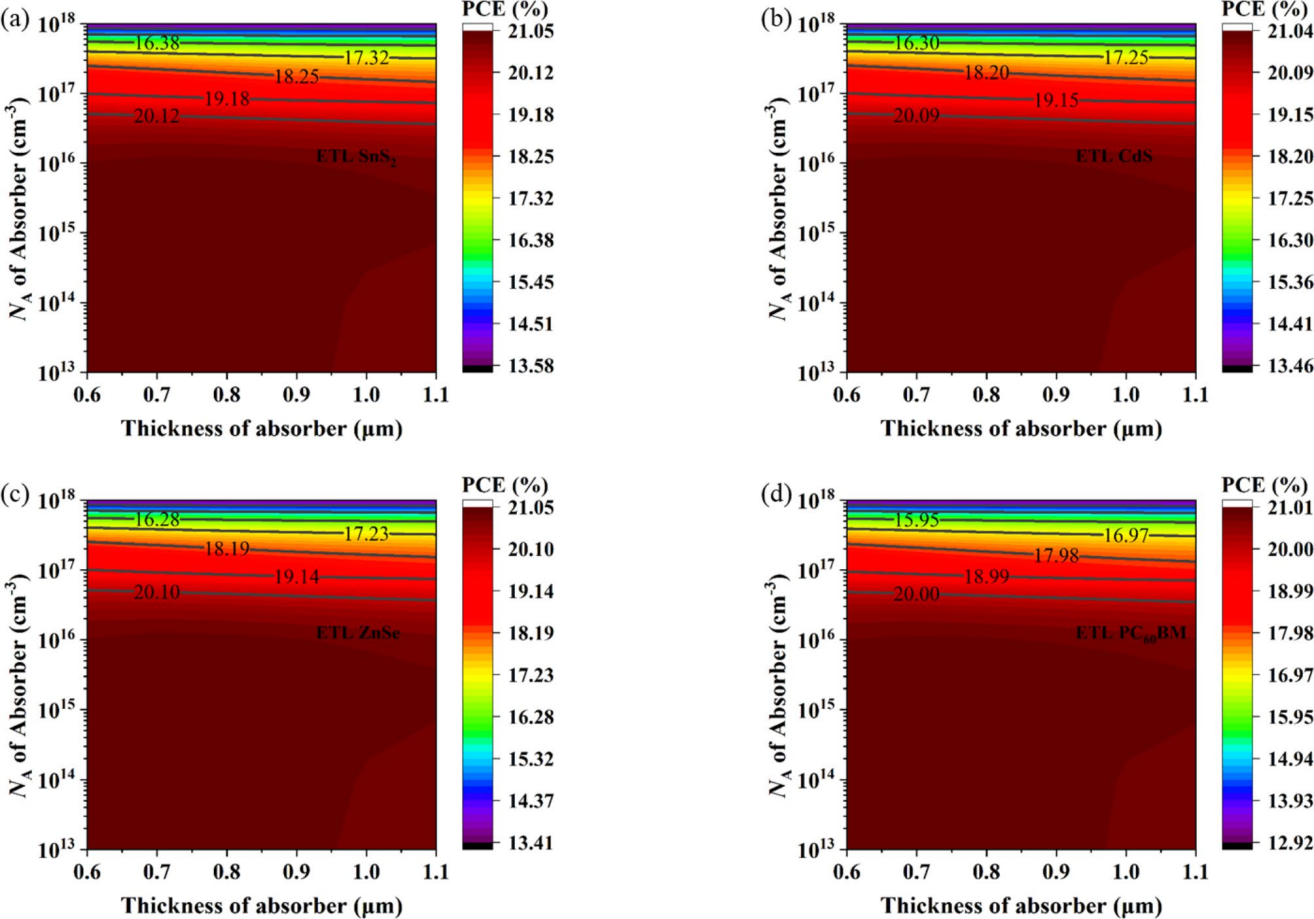
Correlation between absorber acceptor density and absorber layer thickness.

The absorber layer thickness and acceptor are critical factors impacting device performance. Optimizing these parameters is necessary to get optimal performance. During simulation, the absorber thickness was adjusted from 0.5 μm to 1.5 μm, and the N_A_ was modified from 1 × 10^13^ cm^− 3^ to 1 × 10^20^ cm^− 3^ to examine the effects of these factors on the PV performance characteristics of the four optimized PSCs. The impact of simultaneously varying the thickness of the absorber layer and N_A_ using contour plot mapping is seen in Fig. 9 on the PCE for the buildings under investigation. It is pleasing to note that when the thickness of the absorber was modified using absorber N_A_, SnS_2_, PC_60_BM, ZnSe, and CdS ETL-based PSC structures displayed almost similar patterns. When both the NA and absorber thickness are less extensive than 1 × 10^16^ cm^− 3^ and 0.95 μm, respectively, the maximum PCE (~ 21.05%) was observed in these four solar structures. Additionally, ZnSe and CdS as the ETL have the most prominent PCE among the four optimized PSCs, whereas PC_60_BM exhibits the lowest PCE. Therefore, it can be said that for CsPb._625_Zn._375_IBr_2_ absorber-based PSCs, it is better to employ inorganic oxide-based ETLs rather than organic ones.

### Effect of absorber layer thickness with absorber donor density

**Fig. 10:**
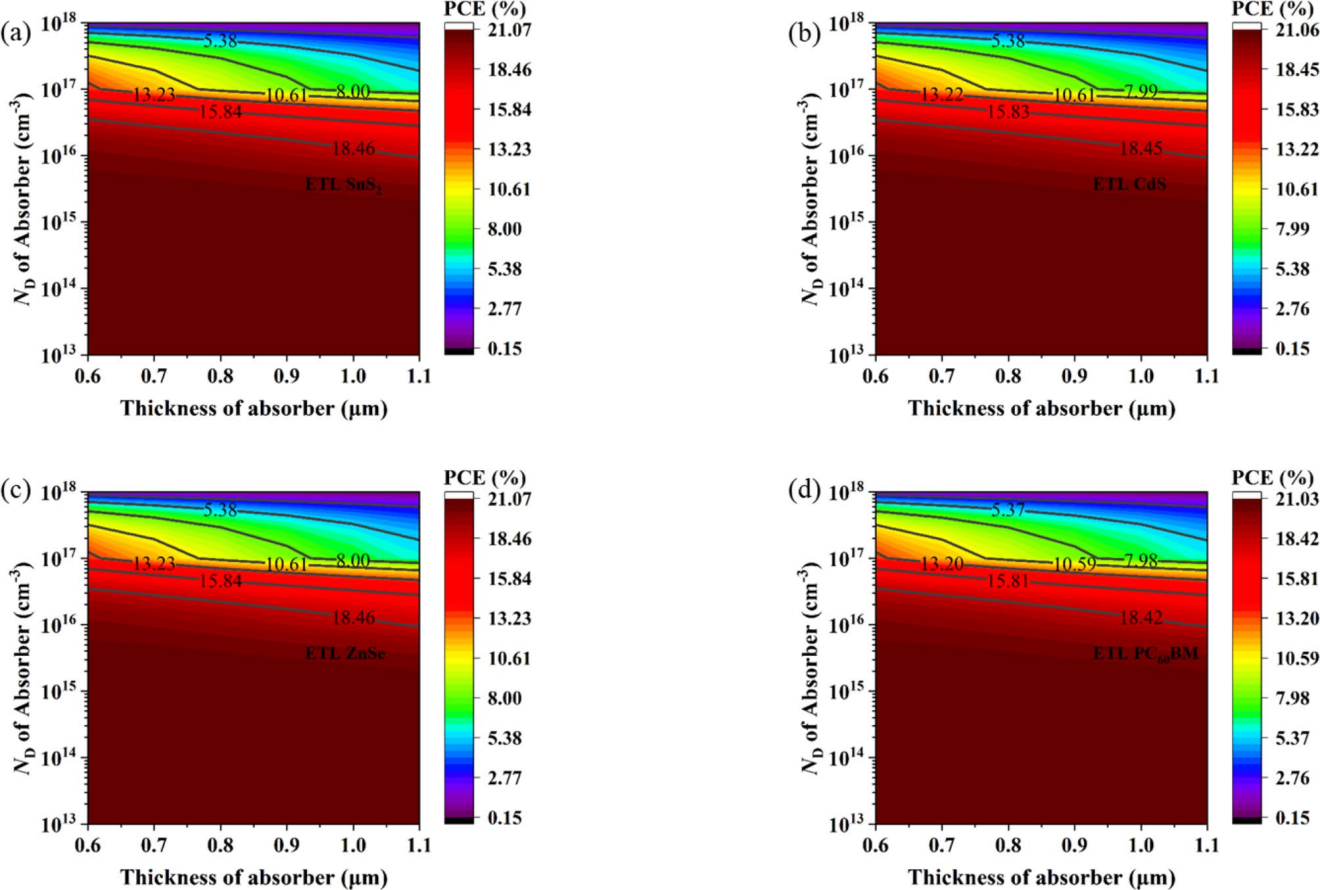
Effect of absorber layer thickness with absorber donor density.

This work uses contour mapping to examine how adjusting the absorber donor density and width of the absorber layer affects the performance of the CsPb._625_Zn._375_IBr_2_ absorber-based PSCs. When N_D_ is less than 1 × 10^16^ cm^− 3^ and the absorber thickness is 0.6 μm to 1.1 μm, the PCE is at its highest (> 21%). When N_D_ rises more than 1 × 10^16^ cm^− 3^, PCE starts to fall as well, but absorber layer thickness has no impact on PCE. As Fig. 10 shows, the maximum value of PCE (21.07%) can be obtained using SnS_2_ and ZnSe ETL-based PSC structures whereas PC_60_BM exhibits the lowest PCE. Therefore, it can be said that for CsPb._625_Zn._375_IBr_2_ absorber-based PSCs, it is better to employ inorganic oxide-based ETLs rather than organic ones.

### Effect of absorber layer thickness with absorber defect density

**Fig. 11:**
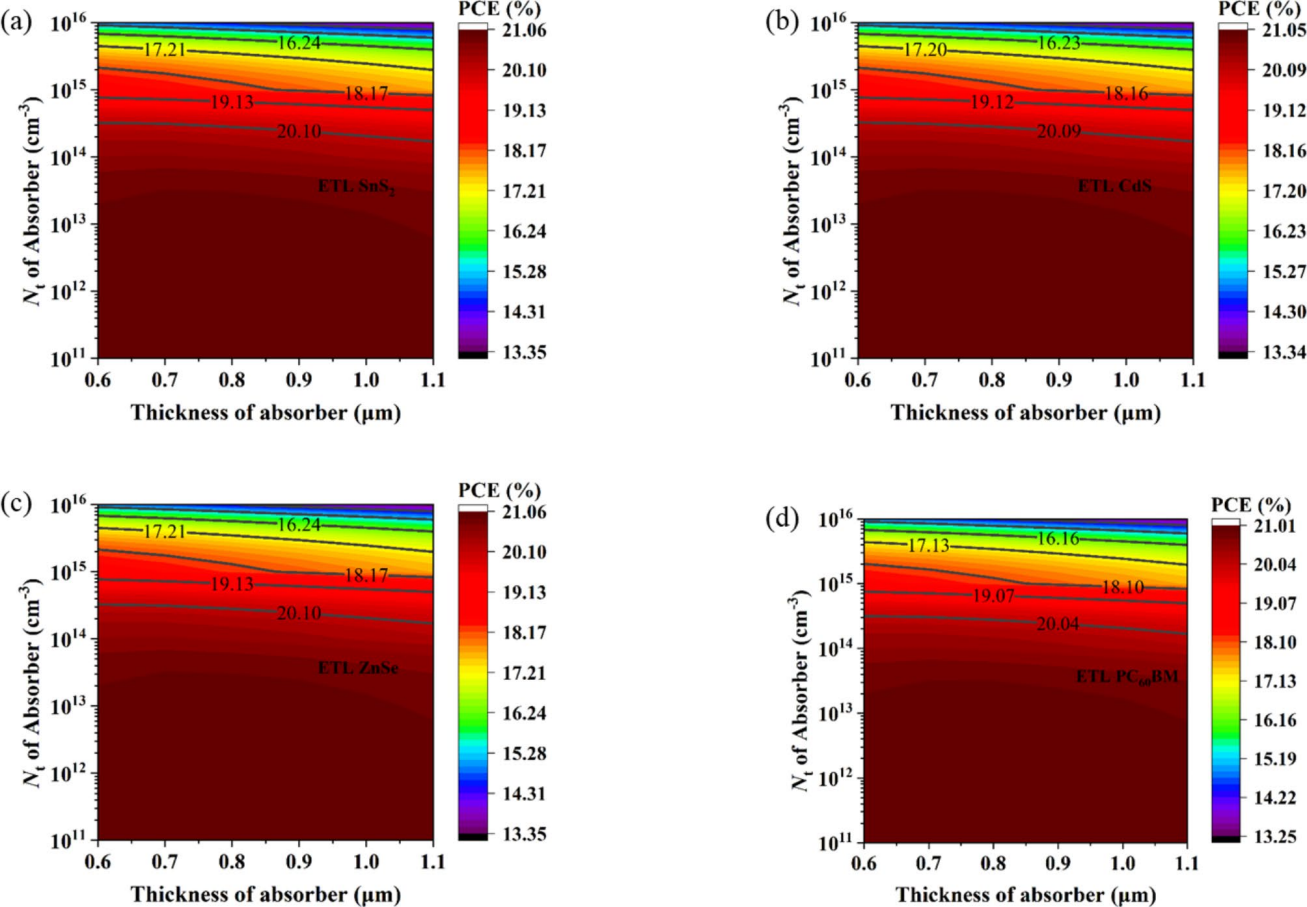
Effect of absorber layer thickness with absorber defect density.

The absorber thickness and defect density have a major direct impact on the Solar cell performance. Increased recombination lowers the PCE of PSCs because greater N_t_ in the absorber layer causes pinhole formation and film breakdown^30,110^. By adjusting the absorber depth between 0.6 μm and 1.1 μm, the simulation was run to determine the most beneficial defect density for a given absorber layer thickness and N_t_ adjusted between 1 × 10^11^ cm^− 3^ to 1 × 10^16^ cm^− 3^. For every building under investigation, Fig. 11 shows variations in absorber layer thickness and N_t_, which cause PCE to fluctuate. Figure 11 illustrates how variations in absorber thickness and N_t_ affect PCE. As ETL-based solar structures, SnS_2_, PC_60_BM, ZnSe, and CdS exhibit a comparable pattern for the PCE value with adjustments to N_t_ and absorber thickness, as shown in Fig. 11. Out of all the devices under study, SnS_2_ and ZnSe, as the ETL demonstrated the maximum PCE of around 21.05% happens when the absorber depth ranges from 0.6 μm to 1.1 μm and defect density, is less than 2 × 10^13^ cm^− 3^. In the presence of a defect, inorganic ETLs (SnS_2_, ZnSe, and CdS) based PSCs outperform the organic ETLs-based PSCs.

### Effect of generation rate and recombination rate

**Fig. 12:**
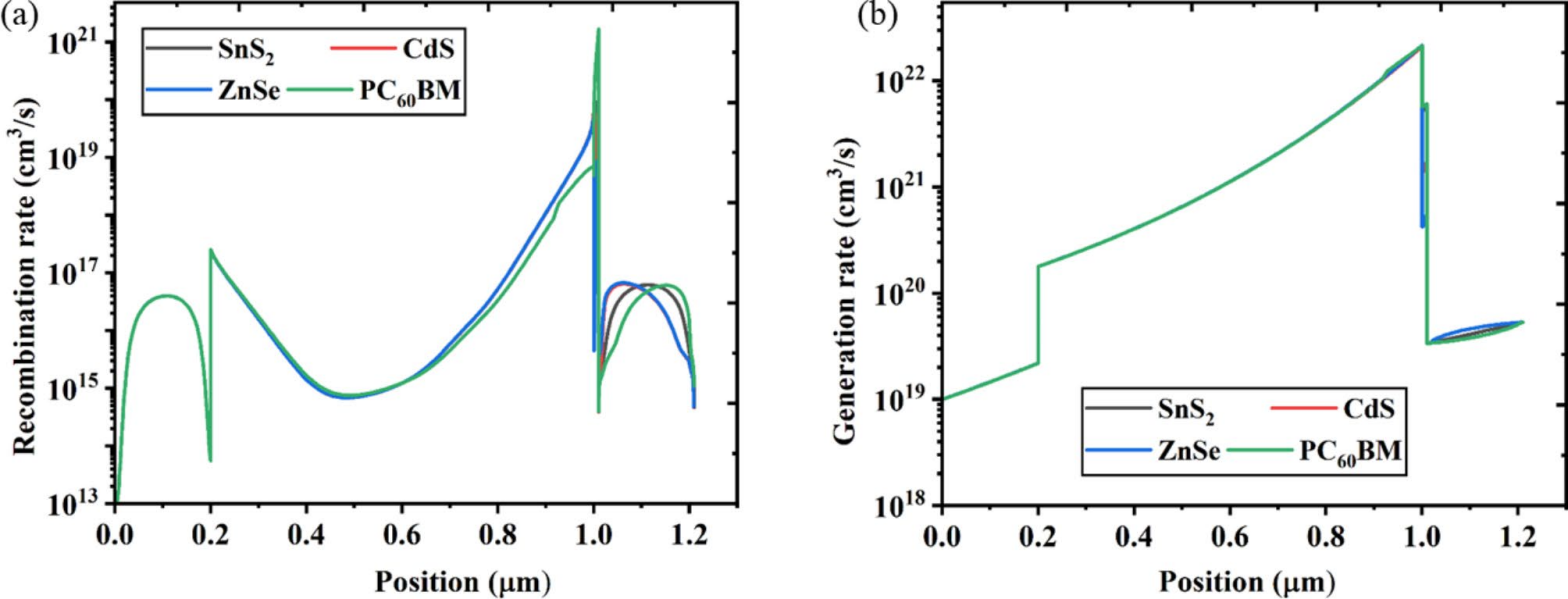
Effect of generation rate and recombination rate.

The coupling and annihilation of electrons and holes in the conduction band is the process of recombination. The lifespan and charge carrier density both have an impact on the recombination rate. Furthermore, the electron-hole recombination is affected by every defect condition present in the several PSC layers^95^. The four PSCs had a maximum recombination rate that was like the generation rate, within 1.0 μm, as seen in Fig. 12a. The region between 1.0 μm displayed an elevated recombination rate due to more conduction band electrons crossing the energy barrier, entering the valence band, and becoming more stable by taking the position of the valence band hole. The consequence of energy levels influences the electron hole’s recombination rate inside the device, and imperfection and grain boundaries may cause the recombination rate distribution in the solar architecture to be non-uniform^95^.

The generated electron-hole pair may be found for any wavelength of light, the whole conventional solar spectrum, and at any position within the solar cell. The largest generation rate is near the device surface, where most of the light is absorbed^30,110^. The generation rate of a solar cell indicates the quantity of electron-hole pairs produced inside the device as a result of photon absorption at a certain wavelength., which depends on both position and wavelength. The generation rate is essential for PSCs to operate as efficiently as possible. The four optimized solar devices’ generating rates are displayed in Fig. 12b. As the figure makes clear, all four different structure PSCs based on SnS_2_, ZnSe, PC_60_BM, and CdS ETLs demonstrated generation rates start increasing from 0.2 μm and reached greater generation rate at PSC depths of 1.0 μm.

### JV and QE characteristics curve

**Fig. 13:**
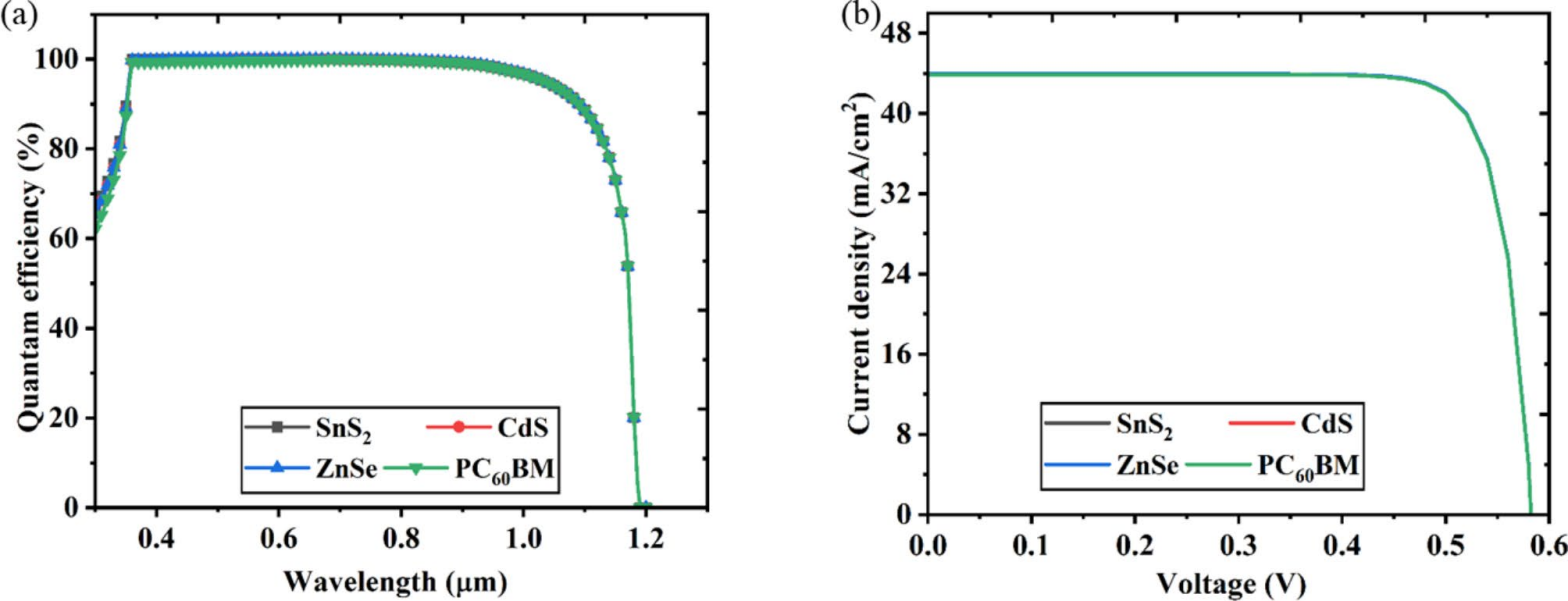
JV and QE characteristics curve.

The quantity of current produced by photoirradiation at a certain wavelength is known as a perovskite solar cell’s quantum efficiency (QE)^118,119^. The number of charge carriers that are moved and gathered by the electrodes can also be used to calculate the QE. The ideal form of a solar cell’s QE curve is square or rectangular; however, it can be distorted by factors such as surface passivation, recombination, and reflection losses^49,103^. For the solar cell architectures of FTO/SnS_2_/CsPb._625_Zn._375_IBr_2_/MoS_2_/Au, FTO/ZnSe/CsPb._625_Zn._375_IBr_2_/MoS_2_/Au, FTO/CdS/CsPb._625_Zn._375_IBr_2_/MoS_2_/Au and FTO/PC_60_BM/CsPb._625_Zn._375_IBr_2_/MoS_2_/Au, the nature of QE for varying wavelength is investigated to more precisely recognize the accumulation of charge carriers. The QE curves for both solar cell architectures are displayed in Fig. 13a before the optimization condition. Figure 13a illustrates that all four structures have a QE of 100% at 400 nm. However, QE begins to decline after 1000 nm wavelength in all cases of structures. The QE graph is square and deemed excellent when the QE value remains constant over the observed wavelength range. The QE of the PSC is lowered due to the recombination, even if the charge carriers cannot go into an external circuit. For most solar cells, recombination lowers QE while charge carriers are incapable of an external circuit. The same factors that affect collection probability also affect QE. Modifying the front surface, for instance, may affect carriers formed close to the surface. Longer wavelength QE can be decreased by the absorbance of free carriers from front surface layers that are highly doped.

The J-V characteristic curve of the PSCs architectures including FTO/SnS_2_/CsPb._625_Zn._375_IBr_2_/MoS_2_/Au, FTO/ZnSe/CsPb._625_Zn._375_IBr_2_/MoS_2_/Au, FTO/CdS/CsPb._625_Zn._375_IBr_2_/MoS_2_/Au and FTO/PC_60_BM/CsPb._625_Zn._375_IBr_2_/MoS_2_/Au are shown in Fig. 13b. As seen in Fig. 13b, all four PSC structures displayed a J_SC_ of about 44 mA/cm^2^ when the V_OC_ was around 0.58 V. Defect states in perovskite films led to a considerable drop in all photovoltaic metrics. This is in line with studies showing that perovskite’s notable crystallization improves its functioning and diminishes charge recombination.

## Conclusion

This work thoroughly examines several CsPb._625_Zn._375_IBr_2_ -based PSC features with varying HTLs and ETLs. Through an examination of key variables influencing PSC performance, the study seeks to identify the optimal design for maximizing conversion efficiency. According to the study, the following elements are crucial for maximizing PSC performance:

1. First of all, we analyzed different types of HTL materials for CsPb._625_Zn._375_IBr_2_ -based PSC, and the best HTL materials for CsPb._625_Zn._375_IBr_2_ -based PSC is MoS_2_.

2. Device performance is significantly impacted by the thickness of the absorber, ETL, and HTL. We found that 800 nm thick absorber layer, 100 nm thick ETL layer, and 50 nm thick HTL layer help us to get the best result.

3. To get the optimum performance we need to set both the acceptor density and donor density of the absorber to less than 1 × 10^16^ cm^-3^ and defect density should not be higher than 1 × 10^14^ cm^-3^.

4. The density of HTL acceptors and ETL donors should be less than 1 × 10^17^ cm^-3^ and the defect density should be less than 1 × 10^15^ cm^-3^.

5. Shunt resistance enhances PCE and FF and has negligible effects on J_SC_ and V_OC_, while an increase in series resistance drastically reduces PCE.

In summary, our thorough analysis shows that several factors, including properties of the ETL and HTL, the width of the absorber layer, acceptor density, and defect densities, influence the performance of CsPb._625_Zn._375_IBr_2_ -based PSCs. By optimizing these parameters, PCEs of 21.05% for the FTO/ZnSe/CsPb._625_Zn._375_IBr_2_/MoS_2_/Au structures and 21.04% for the FTO/CdS/CsPb._625_Zn._375_IBr_2_/MoS_2_/Au structures are obtained. By laying the groundwork for future improvements in stability and efficiency, these optimized designs prepare the ground for using CsPb._625_Zn._375_IBr_2_ -based PSCs in renewable energy applications.

## Data Availability

The datasets used and/or analysed during the current study available from the corresponding author on reasonable request.
